# Green HPTLC–densitometry for simultaneous determination of antibiotic residues in milk with greenness assessment and application to real samples

**DOI:** 10.1038/s41598-026-60367-3

**Published:** 2026-07-05

**Authors:** Sherin F. Hammad, Rawda A. Abozeid, Samar H. Elagamy

**Affiliations:** Department of Pharmaceutical Analytical Chemistry, Faculty of Pharmacy, Tanta University, Tanta, Egypt

**Keywords:** Cefazolin, Sulfadimidine, Marbofloxacin, Greenness, HPTLC, Biochemistry, Biological techniques, Biotechnology, Chemistry, Environmental sciences

## Abstract

A validated HPTLC densitometric approach was employed for the simultaneous determination of Cefazolin (CFZ), Sulfadimidine (SDD), and Marbofloxacin (MFC) residues in milk. The method utilizes a mobile phase composed of ethyl acetate, methanol, and triethylamine in a 50:30:1 v/v/v ratio, with detection carried out at 270 nm. The chromatographic conditions were carefully optimized, and the retardation factor (R_f_) values for CFZ, MFC, and SDD were 0.29, 0.58, and 0.73, respectively. Sample preparation included protein precipitation with acetonitrile, followed by dispersive solid-phase extraction (dSPE) using Enhanced Matrix Removal-Lipid (EMR-L) tubes to remove interfering lipids. Validation in accordance with ICH Q2(R2) guidelines demonstrated excellent linearity for all analytes over the concentration range of 0.001–0.09 µg/band. Accuracy was confirmed by recovery values between 98.19% and 99.15%. The proposed method was successfully applied to the determination of the investigated drug residues in real cattle milk samples following the withdrawal period, showing no statistically significant differences in accuracy and precision compared to previously reported HPLC method. Furthermore, sustainability evaluation using multiple greenness assessment tools (Eco-scale score of 77, AGSA score of 75%, BAGI score of 77.5, and RGB12 score of 85.2) demonstrated that the method is excellent green.

## Introduction

Monitoring veterinary drug residues in milk is crucial for food safety, as their widespread use in dairy cattle can lead to harmful levels that pose risks such as antimicrobial resistance, allergic reactions, and disruption of human gut health^1^. To mitigate these risks, regulatory authorities set Maximum Residue Limits (MRLs), which defined as the highest permissible levels of pharmacologically active substances in animal-derived foods^2–4^. High-Performance Thin-Layer Chromatography (HPTLC) densitometry is a valuable method for screening and quantifying veterinary drug residues in milk to ensure compliance with MRLs^5^. HPTLC-densitometry offers high throughput, enabling simultaneous processing of multiple samples and standards on a single plate, reducing analysis time and cost per sample^5^. Although less sensitive than HPLC, HPTLC–densitometry is a greener analytical technique characterized by lower energy requirements and reduced solvent consumption^6^.

This study investigates three commonly used antibiotics in milk— marbofloxacin (MFC), cefazolin (CFZ), and sulfadimidine (SDD) which are often administered in combination for the treatment of bovine metritis. Metritis is a postpartum uterine infection characterized by inflammation and foul-smelling discharge, resulting from bacterial contamination during or after calving and negatively impacting reproductive efficiency and milk production^7^.

**Fig. 1:**
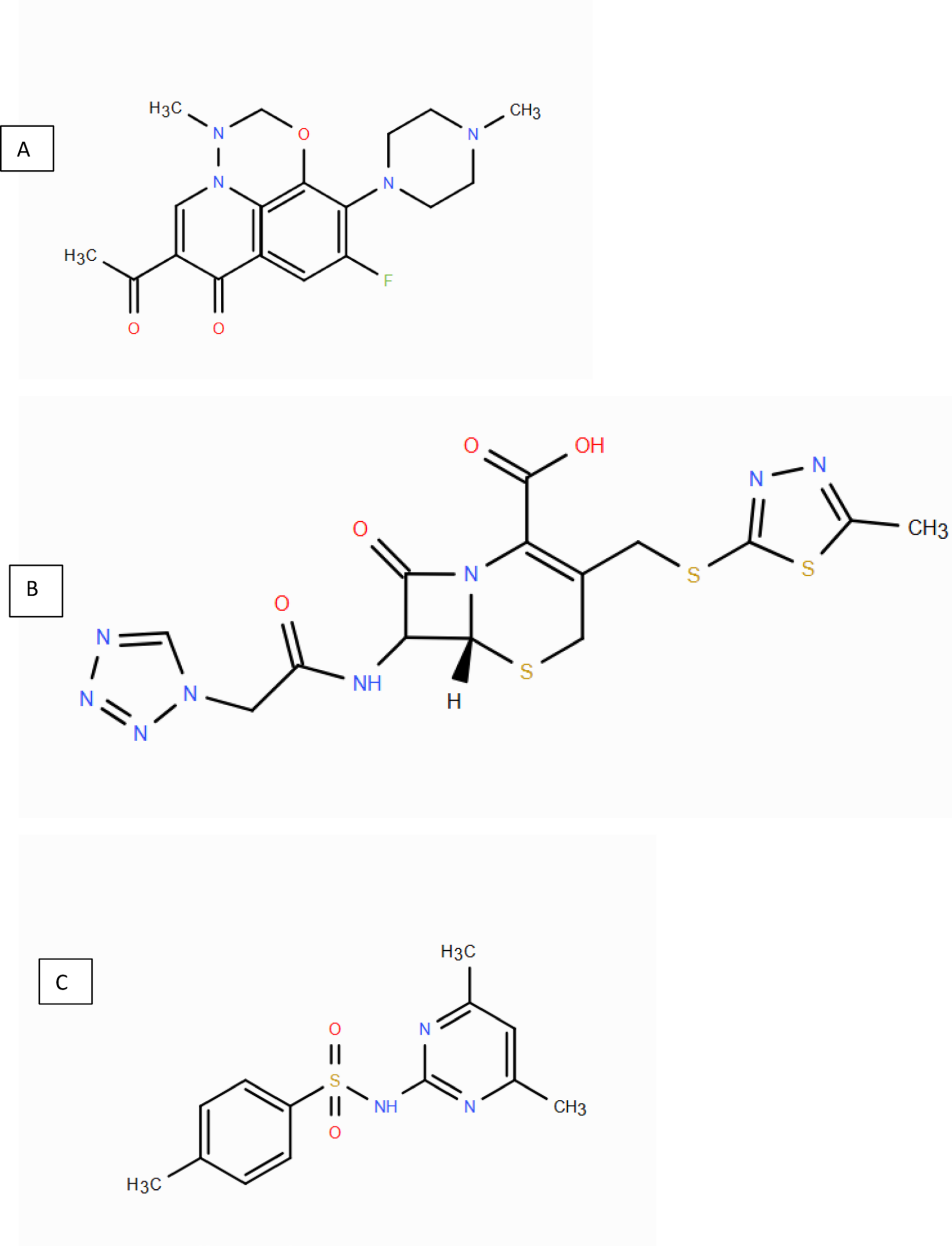
Chemical structures of (**A**) MFC, (**B**) CFZ, and (**C**) SDD.

**MFC (**Fig. 1a**)**, chemically known as 9-fluoro-2,3-dihydro-3-methyl-10-(4-methyl-1-piperazinyl)−7-oxo-7 H-pyrido[1,2,3-de][1,4]benzoxazine-6-carboxylic acid, is a fluoroquinolone antibiotic that acts by inhibiting DNA and RNA synthesis. It is extensively used in veterinary medicine for the treatment of a wide range of bacterial infections in animals. It is particularly effective against skin and soft tissue infections, urinary tract infections, respiratory tract infections, and other bacterial conditions^8^. Several analytical methods have been reported for the determination of MFC in milk, such as HPLC^8–16^, and LC-MS/MS^17–23^.

**CFZ (**Fig. 1b**)**, is a cephalosporin antibiotic, chemically known as (6R,7R)−3-[(5-methyl-1,3,4-thiadiazol-2-yl)thiomethyl]−7-[(1H-tetrazol-1-yl)acetamido]-oxo-5-thia-1-azabicyclo[4.2.0]oct-2-ene-2-carboxylic acid, primarily used for the treatment of bacterial skin infections, severe systemic infections, and urinary tract infections. This bactericidal agent acts by binding to penicillin-binding proteins (PBPs), thereby blocking the final transpeptidation step of peptidoglycan formation in cell wall synthesis. It is mainly effective against Gram-positive aerobic bacteria. However, its activity against Gram-negative organisms is limited, and it shows poor efficacy against anaerobic bacteria. Various methods have been reported for determining CFZ levels in milk, including TLC^24,25^, HPLC^26–31^, and LC-MS/MS^32–40^.

**SDD (**Fig. 1c**)**, chemically known as 4-amino-N-(4, 6-dimethylpyrimidin-2-yl) benzenesulfonamide, inhibits bacterial growth by competitively blocking the enzyme dihydropteroate synthase. It interferes with folic acid synthesis, thereby preventing the production of essential nucleic acids required for bacterial growth and replication. It is widely used in veterinary medicine to treat bacterial and protozoal infections in livestock^41^. Several analytical techniques have been reported for the determination of SDD in milk, including TLC^42,43^, LC-MS/MS^40–50^, and HPLC^45–54^.

A review of the available literature for determination of these antibiotics revealed that LC–MS/MS methods have been reported for their determination in combination with other antimicrobial classes in feed samples^55^, as well as in beef, pork, and fish tissues^56^. Also, an HPLC method with UV detection has been previously reported for the determination of these antibiotics in milk samples^57^; however, no HPTLC method has yet been described for their simultaneous analysis in milk. Compared to HPLC and LC-MS/MS, HPTLC offers distinct advantages: (i) reduced solvent consumption (milliliters vs. liters per analysis), (ii) lower energy requirements (no high-pressure pumps), (iii) simpler sample preparation, (iv) lower instrumentation and operational costs, (v) shorter analysis time per sample when multiple samples are run simultaneously, and (vi) simultaneous processing of up to 20 samples on a single plate. In contrast, HPLC methods typically require 30–60 min per sample with complex mobile phase gradients, while LC-MS/MS demands expensive instrumentation and specialized technical expertise. Accordingly, the present study aims to develop and validate a sustainable HPTLC–densitometric method for the combined determination of these antibiotics in real milk samples.

In milk sample analysis, proper sample preparation is essential to remove interfering substances and ensure accurate analyte determination due to the complexity of the matrix. Although traditional techniques such as liquid–liquid extraction are widely used, they suffer from high solvent consumption, low selectivity, and labor-intensive procedures, while protein precipitation offers a simpler and more cost-effective alternative but often requires additional cleanup because of the high lipid content of milk. To address this challenge, dispersive solid-phase extraction techniques, including Agilent’s Enhanced Matrix Removal–Lipid (EMR-Lipid), are employed to efficiently remove fats while preserving analyte recovery and improving extract cleanliness^68–70^.

## Experimental

### Apparatus

Chromatographic separation was performed on TLC aluminum plates (20 × 20 cm, 0.2 mm) pre-coated with silica gel 60 F254 purchased from Merck (Darmstadt, Germany). Samples were applied using a CAMAG Linomat V automatic sample applicator (Muttenz, Switzerland) with a 100-µL microsyringe. The bands were visualized using a UV lamp with a short wavelength of 254 nm (SPECTROLINER Model ENF-260 C, U.S.A.) Densitometric scanning of the obtained bands was carried out using a CAMAG TLC Scanner III (Camag, Switzerland), operating in absorbance mode and controlled by WinCATS software, version 1.4.1 (Camag, Switzerland). The scanning speed was 20 mm/s with a slit dimension of 6.0 mm × 0.3 mm, and a data resolution of 100 nm/step. Densitometric analysis of chromatograms was performed at 270 nm (± 1 nm). A Bio-Base PHS-3BW precision pH meter and Bio-Base BTFC-12MRT3 high-speed centrifuge were utilized for sample preparation.

### Materials

#### Chemicals and reagents

Marbofloxacin (MFC, 99.99% purity), Cefazolin (CFZ, 98.86% purity), and Sulfadimidine (SDD, 99.37% purity) were supplied by Pharma-Swede Company (Egypt). Acetonitrile and methanol were purchased from Honeywell, while ethyl acetate was obtained from Sigma-Aldrich. Ultrapure water (LiChrosolv grade) was used throughout the study. Triethylamine and glacial acetic acid were supplied by EL-NASR Chemicals. For dispersive solid-phase extraction, Bond Elut EMR-Lipid d-SPE tubes were obtained from Agilent Technologies (Germany).

#### Milk samples

Five independent fresh raw milk samples were collected from five different lactating bovines (single sampling per animal; three buffaloes and two cows) with an average age of approximately 2 years and a body weight of 525 ± 28 kg. The animals were maintained under a tie-stall housing system on a farm located in Sharqia Governorate, Egypt. All selected animals were diagnosed with metritis and were under combination therapy of the studied antibiotics at their recommended therapeutic doses as prescribed by a veterinarian. Milk samples (25 mL) were collected aseptically by a veterinarian after a 72-hour withdrawal period using sterile 50 mL Falcon tubes, then stored at − 20 °C until analysis. All sampling procedures were conducted in accordance with institutional and national ethical guidelines for animal care and use^58^. The samples were filtered through 0.45-µm nylon membrane filters to eliminate the suspended matter.

#### Standard solutions

Stock solutions of MFC, CFZ, and SDD were prepared individually by dissolving 25 mg of each drug in 250 mL of distilled water to obtain a concentration of 100 µg/mL. Working standard solutions (10 µg/mL) were freshly prepared by appropriate dilution of 5 mL of stock solution to 50 mL with water.

### Procedures

#### Samples preparation

**Fig. 2:**
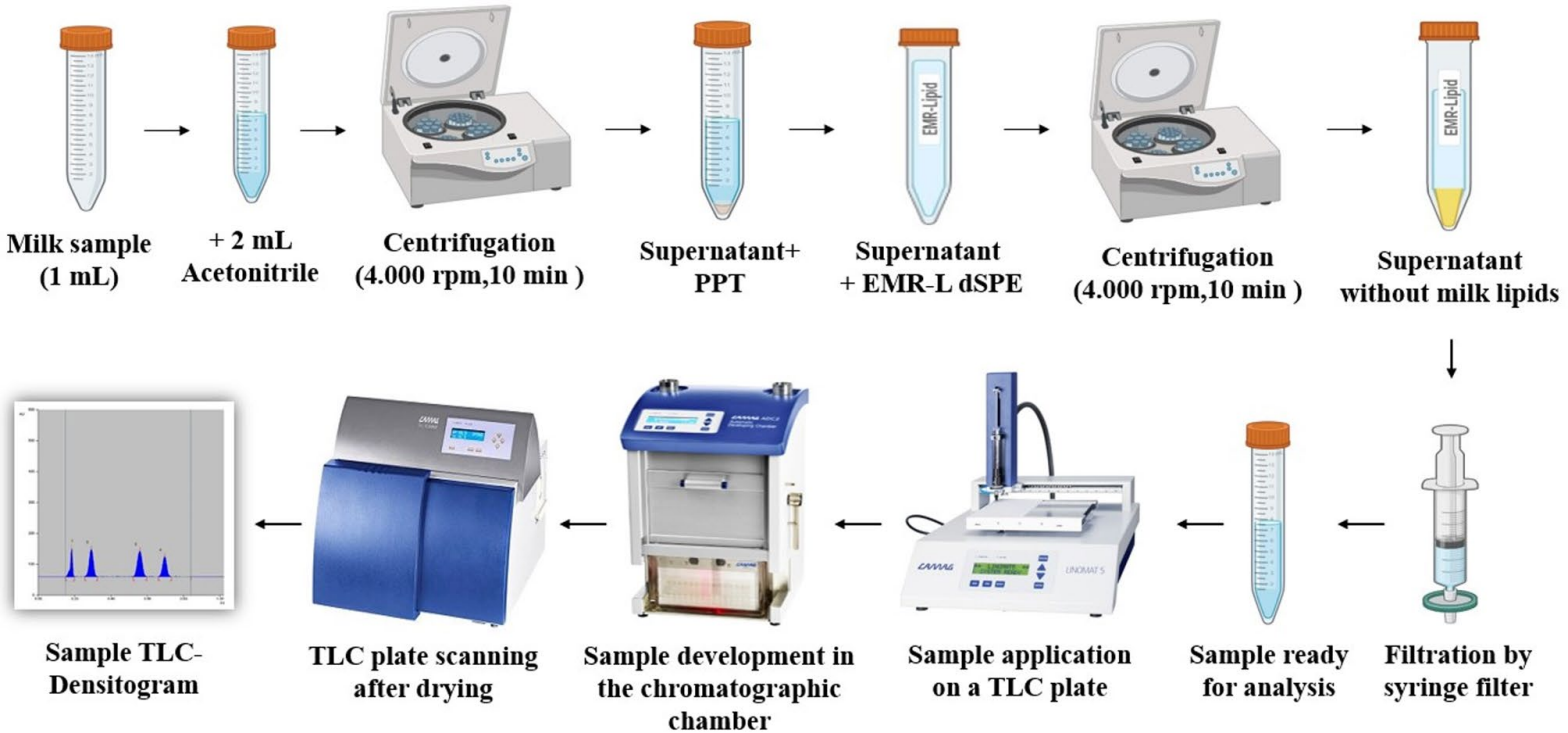
Flow chart illustrating the sample preparation and chromatographic analysis procedures.

A 1.0 mL portion of milk (≈ 1 g) was placed in a 10 mL centrifuge tube, followed by the addition of 2.0 mL of acetonitrile to precipitate proteins. The mixture was vortexed and centrifuged at 4,000 rpm for 10 min, and the resulting supernatant was collected. This extract was then subjected to lipid removal using a 15 mL EMR-Lipid dispersive solid-phase extraction tube, which was activated and centrifuged again at 4,000 rpm for 10 min, following the procedure reported by Hammad et al.^57^. The clarified supernatant obtained after cleanup was filtered and transferred to a clean tube for HPTLC analysis. The overall sample preparation workflow is presented in Fig. 2.

#### Chromatographic conditions

Samples were applied as bands on TLC aluminum plates (20 × 10 cm, 0.20 mm) pre-coated with silica gel 60 F254 by CAMAG LINOMATE V automatic applicator using 100 µL Hamilton micro-syringe. The bandwidth was adjusted to be 6 mm. Each band was spaced 1 cm apart from each other and 1 cm from the bottom edge of the plate. The chromatographic chamber was pre-saturated with the mobile phase for 25 min. The plates were developed to a distance of approximately 8 cm by ascending technique using ethyl acetate: methanol: triethylamine (50:30:1, v/v/v) as a mobile phase. Following development, the plates were dried in air at room temperature and scanned at 270 nm using CAMAG TLC scanner III, operating in the absorbance mode with a deuterium lamp as a source of radiation. The slit dimension was kept at 3 mm x 0.45 mm and scanning speed was employed to be 20 mm/s. The chromatographic analysis is illustrated in Fig. 2.

#### Validation of the procedures

##### Construction of calibration curves (linearity)

Standard solutions were prepared by transferring measured volumes (0.1–9 mL) from the 10 µg/mL stock solutions of MFC, CFZ, and SDD and into 100 mL volumetric flasks. After dilution to the mark with distilled water and complete mixing, the resulting solutions covered a concentration range of 0.01–0.9 µg/mL. Aliquots of 100 µL from these solutions were applied to TLC plates to achieve concentrations ranging from 0.001 to 0.09 µg/band for all the studied drugs. This concentration range was across the specified MRL of each drug. The MRLs for MFC CFZ, and SDD are 0.015, 0.005, 0.01 µg/band, respectively. Drug concentrations were calculated using linear calibration plots of peak area (270 nm) versus concentration.

##### Accuracy and precision evaluation

The trueness (accuracy) and repeatability (precision) of the proposed method were evaluated by analyzing three concentration levels of each analyte (0.005, 0.04, and 0.09 µg/band) in triplicate. Intra-day precision was assessed through repeated measurements within a single chromatographic run, while inter-day precision was determined across three independent runs performed on three consecutive days. Accuracy was expressed as percentage recovery along with standard deviation, whereas precision was evaluated in terms of relative standard deviation (RSD %).

##### Assessment of extraction efficiency

The efficiency of the extraction procedure was determined by comparing the chromatographic responses of spiked milk samples at three concentration levels (0.005, 0.04, and 0.09 µg/band) with those obtained from equivalent standard solutions prepared in water. Although absolute recovery of 100% is not mandatory, the method should ensure consistent and reproducible recovery values across the studied concentration range, with acceptable precision.

##### Specificity and selectivity assessment

Method specificity was evaluated by analyzing five blank milk samples processed using the same extraction procedure and chromatographic conditions. These samples included three buffaloes and two cows, collected from different sources. The purpose was to investigate potential interferences from endogenous milk constituents and confirm that no co-eluting peaks affected the detection of the target analytes.

##### Assay of real samples

Milk samples were collected individually from five independent lactating, metritis-positive cattle after the withdrawal period for each drug (72 h)^16,31,46^. All animals received the final intramuscular dose of the studied drugs simultaneously under veterinary supervision. After 72 h, a single milk sample (25 mL) was collected from each animal and processed using the optimized sample preparation protocol. From each prepared extract, 100 µL aliquots were applied as bands on the TLC plates. Following chromatographic development under the established conditions, densitometric scanning was performed at 270 nm, and peak areas were recorded. The concentrations of the analytes (µg/band) were calculated using their corresponding calibration equations.

## Result and discussion

This study aimed to develop a reliable and precise HPTLC-densitometric method for the simultaneous quantification and routine quality control of CFZ, SDD, and MFC residues in milk samples. To ensure accurate determination, we optimized the extraction process and mobile phase composition, minimizing potential interference from impurities.

###  Optimization of experimental conditions

The studied antibiotics are selected for their complementary mechanisms of action, broad-spectrum efficacy, and ability to reduce resistance development while improving therapeutic outcomes^59–61^. These drugs are excreted unchanged in milk, and their residues can pose potential health risks to consumers^16,31,46^. The European Union (EU) has set MRL for MFC, CFZ, and SDD in milk at 150, 50, and 100 µg/kg (equivalent to 0.15, 0.05, and 0.1 µg/ml), respectively^62,63^.

The TLC chromatographic separation was systematically optimized through a trial-and-error approach to achieve the maximum resolution of the target analytes from each other and from the co-extracted polar milk matrix impurities. Various mobile phases with different compositions and solvent ratios, ranging in polarity, were tested to obtain satisfactory separation. By using standard silica gel 60 F₂₅₄ plates, a non-polar mobile phase of chloroform was initially tried and resulted in no migration (R_f_ = 0) for all analytes and polar matrix impurities. Then a mobile phase consisting of chloroform and methanol (90:10, v/v) was tried and resulted in poor resolution of the studied drugs. CFZ (R_f_ ≈ 0.05) remained close to the baseline, while SDD and MFC co-eluted with R_f_ values of approximately 0.68 and 0.65, respectively. The polar milk matrix impurities were strongly retained at the baseline with the lowest R_f_ value (≈ 0.02) due to their high polarity and minimal affinity for the non-polar mobile phase.

To enhance the separation, the mobile phase polarity was gradually increased to chloroform and methanol (70:30, v/v). This adjustment improved CFZ mobility (R_f_ ≈ 0.11) but still did not provide adequate baseline resolution, while SDD and MFC were still overlapped at R_f_ values of approximately 0.52 and 0.48, respectively. The polar milk matrix impurities still retained at a very low R_f_ value of around 0.04.

By replacing chloroform with ethyl acetate (less non polar solvent) and adding a weak acid modifier such as glacial acetic acid to the mobile phase, to be as follows: ethyl acetate: methanol: glacial acetic acid (60:30:1, v/v/v), MFC (R_f_ 0.35) was successfully separated from SDD (R_f_ 0.55). This was accomplished by reducing the tailing of the acidic MFC. However, CFZ (R_f_ 0.08) and the polar milk matrix impurities (R_f_ 0.03) were still not adequately mobile and poorly resolved from each other.

The optimal separation was finally achieved using a ternary mobile phase of ethyl acetate, methanol, and triethylamine at a ratio of 50:30:1, v/v/v, respectively. This system provided excellent baseline resolution for all components, with distinct and well-defined bands with an elution order as follows: polar milk matrix impurities (R_f_ 0.18), CFZ (R_f_ 0.29), MFC (R_f_ 0.58), and SDD (R_f_ 0.73).

By adding triethylamine as a basic modifier in the mobile phase system, it binds with the acidic silanol groups (Si-O⁻) on the silica gel, preventing cationic analytes from sticking to them, allowing CFZ to mobilize effectively. It also increases the pH of the mobile phase, which helps keep basic compounds in their non-ionized state. For example, the primary amino group in SDD was deprotonated by adding triethylamine in the mobile phase system, leading to the loss of its positive charge and reducing its ionic interaction with the negatively charged silanol groups (Si-O⁻) on the silica gel surface. This drastically reduces its retention, giving it the highest R_f_ value. In contrast, the milk matrix impurities, consisting of highly polar amino acids and sugars, showed the lowest R_f_ value due to their strong hydrophilic interaction with the silica gel and minimal affinity for the non-polar eluent, leading to better chromatographic separation and resolution from the target analytes.

**Fig. 3:**
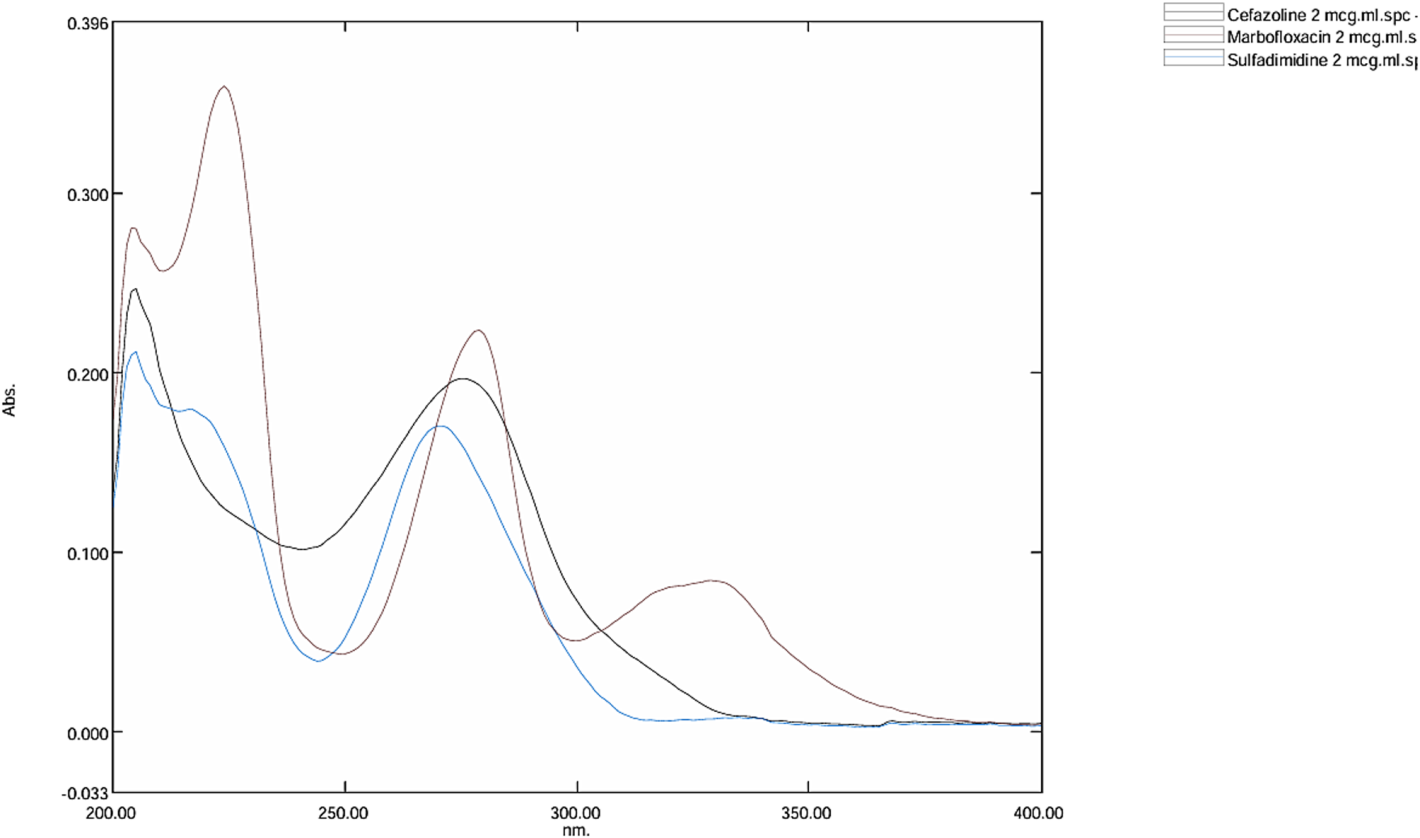
Overlay UV spectra of CFZ, SDD, and MFC.

**Fig. 4:**
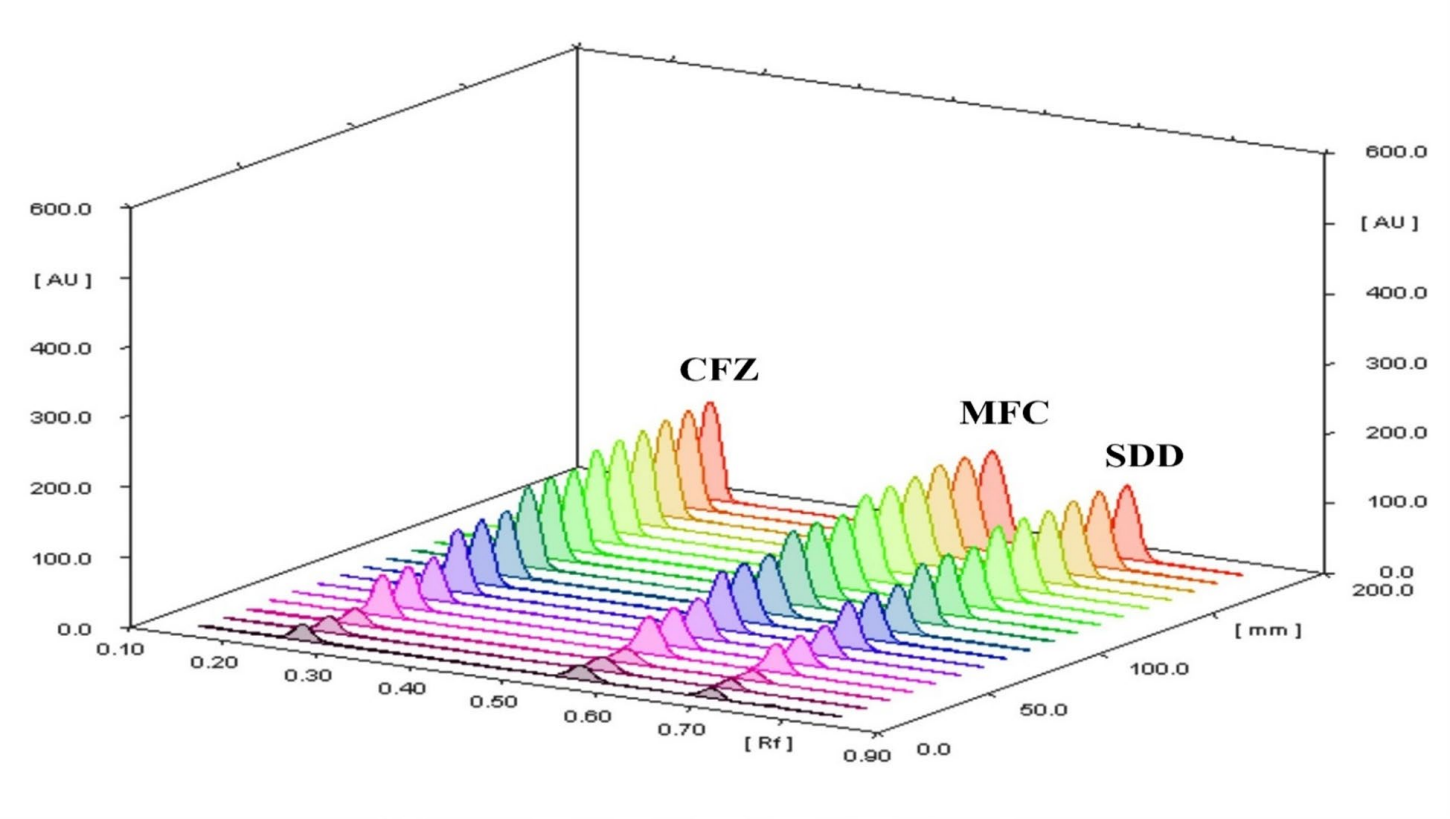
3D-thin layer chromatography-densitogram showing different concentrations of the studied drugs (0.001–0.09 µg/band) at 270 nm.

The scanning wavelength was carefully optimized to achieve the best results. UV detection was performed at 270 nm, corresponding to the λmax of SDD, while ensuring optimal sensitivity for CFZ and MFC, as shown in Fig. 3. The method exhibited excellent reproducibility, with mean Rf values of 0.29 ± 0.02 for CFZ, 0.58 ± 0.04 for MFC, and 0.73 ± 0.03 for SDD, as illustrated in Fig. 4.

A 100 µL application volume is typically selected to balance sensitivity, resolution, and practical throughput on HPTLC plates. This volume is critical for accurately measuring veterinary drug residues in milk, as they must adhere to strict maximum residue limits (MRLs), often at very low levels. By applying a larger volume as narrow bands rather than traditional spots, a sufficient absolute mass of each analyte is deposited within a compact zone. This achieves the necessary signal-to-noise ratio for reliable densitometric quantification without compromising chromatographic resolution or overloading the silica gel layer. Consequently, this workflow eliminates the need for time-consuming offline evaporation, enabling sensitive, high-throughput trace analysis directly from milk extracts.

### System suitability parameters

System suitability testing was conducted to evaluate the performance of the chromatographic system according to USP guidelines^64^. Parameters such as retardation factor (R_f_), resolution (Rs), tailing factor (T), capacity factor (K`), and selectivity factor (α) were assessed. The chromatographic peaks for CFZ, MFC, and SDD displayed symmetric profiles without tailing, as indicated by the tailing factor (T) values. The selectivity factors (α) confirmed sufficient chromatographic separation, while resolution values (Rs) exceeded 1.5 for all analyte pairs, ensuring baseline separation Table 1.

**Table 1:** System suitability parameters of the developed HPTLC-densitometric method.

Parameters	CFZ	MFC	SDD	Acceptance criteria^64^
Retardation factor (Rf)	0.29 ± 0.02	0.58 ± 0.04	0.73 ± 0.03	–
Tailing factor (T)	1.09	0.98	0.99	~ 1
Capacity factor (K`)	1.04	3.76	5.26	1–10 acceptable
SD of peak area	11.42	8.88	3.86	–
Selectivity factor (α) _**CFZ−MFC**_	2.7			α > 1
Selectivity factor (α) _**MFC−SDD**_	2.2			α > 1
Resolution (R_s_) _**CFZ−MFC**_	5.5			Rs > 1.5
Resolution (R_s_) _**MFC−SDD**_	3.2			Rs > 1.5

### Method validation

The validation of the analytical method was performed following the International Council for Harmonization (ICH) Q2 (R2) recommendations^65^, with comprehensive assessment of key validation parameters such as linearity, specificity, accuracy, repeatability, and intermediate precision.

#### Linearity

The calibration curves for the studied drugs were generated by establishing the relationship between the peak area (at 270 nm) and the corresponding drug concentrations (µg/band) under the optimized chromatographic conditions. The regression plots were found to be linear over the range of 0.001–0.09 µg/band for all the studied drugs. This linear concentration range was within the specified MRL of each drug. The MRLs for MFC, CFZ, and SDD are 0.015, 0.005, 0.01 µg/band, respectively. The complete analytical parameters, including the linear ranges, mathematical regression models, y-intercepts, calibration slopes, and correlation coefficients (r), are summarized in Table 2. The strong linear correlation is demonstrated by high correlation coefficient values across all calibration curves.

**Table 2:** Regression parameters for determination of Cefazolin (CFZ), Marbofloxacin (MFC), and Sulfadimidine (SDD) by the proposed HPTLC-densitometric method.

Parameters	CFZ	MFC	SDD
Range (µg/band)	0.001–0.09	0.001–0.09	0.001–0.09
MRL (µg/band)	0.005	0.015	0.01
Regression Parameters	54,500 ± 404.5606	38,669 ± 283.99	27,899 ± 210.569
Slope ± SD			
Intercept ± SD	421.63 ± 5.316	535.55 ± 3.737	355.06 ± 12.187
SD of residuals (Sy/x)	31.304	21.975	16.293
Correlation coefficient (r)	0.9998	0.9998	0.9998
LOD (µg/band)	0.3 × 10^− 3^	0.3 × 10^− 3^	0.29 × 10^− 3^
LOQ (µg/band)	1 × 10^− 3^	1 × 10^− 3^	0.9 × 10^− 3^

#### Detection and quantitation limits

In order to evaluate the sensitivity of the proposed HPTLC densitometric method, the limit of detection (LOD) and limit of quantitation (LOQ) were calculated according to the following equations.

$${\text{LOD }} = {\text{ }}3.3\, \upsigma /{\text{S and LOQ }} = {\text{ }}10\, \upsigma /{\mathrm{S}}$$

Where σ is the standard deviation of the y-intercept of the regression line (*n* = 3), and S is the slope of the calibration curve. The obtained LOD and LOQ values, which are presented in Table 2, confirm the high sensitivity of the developed approach.

#### Accuracy

The recovery rates fell within the range of 99.19% to 100.28%. The accuracy of the method for analyzing ternary mixtures was confirmed by calculating mean percentage recoveries with standard deviations from triplicate determinations, as illustrated in Table 3.

**Table 3:** Results of accuracy for determination of Cefazolin (CFZ), Marbofloxacin (MFC), and Sulfadimidine (SDD) by the proposed HPTLC-densitometric method.

	Conc. (µg/band)	Mean conc. Found ^A^ (µg/band)	% Recovery	Mean % recovery ± SD
CFZ	0.005	0.005	97.79	99.19 ± 1.23
	0.040	0.040	99.66	
	0.090	0.090	100.12	
MFC	0.005	0.005	100.97	100.05 ± 0.81
	0.040	0.040	99.78	
	0.090	0.089	99.40	
SDD	0.005	0.005	101.16	100.28 ± 0.93
	0.040	0.040	100.39	
	0.090	0.089	99.30	

^A^ Average of three determinations.

#### Precision

The calculated percentage relative standard deviation (%RSD) for both repeatability and intermediate precision was found to be less than 2%, as presented in Table 4, demonstrating excellent method reproducibility.

**Table 4:** Evaluation of precision study for determination of Marbofloxacin (MFC), Cefazolin (CFZ), and Sulfadimidine (SDD) by proposed HPTLC-densitometric method.

	Conc. (µg/band)	Intra-day (Repeatability)			Inter-day (Intermediate precision)			Pooled RSD (%)
		Mean conc. Found (µg/band)	Mean % recovery ^A^ ± SD	RSD (%)	Mean conc. Found (µg/band)	Mean % recovery ^A^ ± SD	RSD (%)	
CFZ	0.005	0.005	97.79 ± 0.458	0.469	0.005	98.11 ± 0.297	0.302	0.509
	0.040	0.040	100.43 ± 0.482	0.481	0.040	99.79 ± 0.567	0.568	
	0.090	0.090	99.99 ± 0.434	0.434	0.091	101.06 ± 0.812	0.803	
MFC	0.005	0.005	100.95 ± 0.523	0.518	0.005	100.87 ± 0.125	0.124	0.459
	0.040	0.040	101.15 ± 0.679	0.671	0.040	100.63 ± 0.470	0.467	
	0.090	0.089	99.16 ± 0.811	0.818	0.089	99.00 ± 0.159	0.161	
SDD	0.005	0.005	101.16 ± 0.083	0.082	0.005	100.65 ± 0.527	0.523	0.331
	0.040	0.040	100.25 ± 0.354	0.353	0.040	100.33 ± 0.338	0.337	
	0.090	0.090	100.03 ± 0.407	0.407	0.090	100.33 ± 0.280	0.279	

^***A***^ Average of three determinations.

#### Evaluation of analyte recovery from milk matrix

The analyte recovery (extraction efficiency) was evaluated by comparing the average peak area of the processed samples (0.005, 0.04, and 0.09 µg/band) with those of corresponding non-extracted/authenticated standards. Quantitative analysis revealed extraction recoveries of 98.71 ± 1.25 for CFZ, 99.15 ± 0.87 for MFC, and 98.19 ± 1.99 for SDD, demonstrating excellent recovery rates across the studied concentration range. These results, presented in Table 5, indicate that the extraction methodology provides consistent and efficient recovery of all analytes.

**Table 5:** The extraction efficiency of the proposed sample preparation methodology for the studied drugs.

	Drug Conc. (µg/band)	Average peak area of processed sample	Average peak area of authenticated standard	% Recovery	Average percentage recoveries ± SD
CFZ	0.005	669.37 ± 4.72	688.1 ± 1.25	97.28	98.71 ± 1.25
	0.040	2583.03 ± 5.77	2594.26 ± 10.49	98.14	
	0.090	5294.1 ± 1.73	5332.67 ± 21.31	96.02	
MFC	0.005	717.2 ± 10.44	730.76 ± 1.01	99.57	99.15 ± 0.87
	0.040	2071.06 ± 2.88	2078.96 ± 10.51	99.62	
	0.090	3982.4 ± 1.74	3994.73 ± 3.93	98.60	
SDD	0.005	476.43 ± 2.51	496.16 ± 0.11	99.28	98.19 ± 1.99
	0.040	1454.8 ± 4.58	1475.4 ± 3.93	99.69	
	0.090	2846.9 ± 2.31	2848.43 ± 10.21	99.95	

#### Specificity

**Fig. 5:**
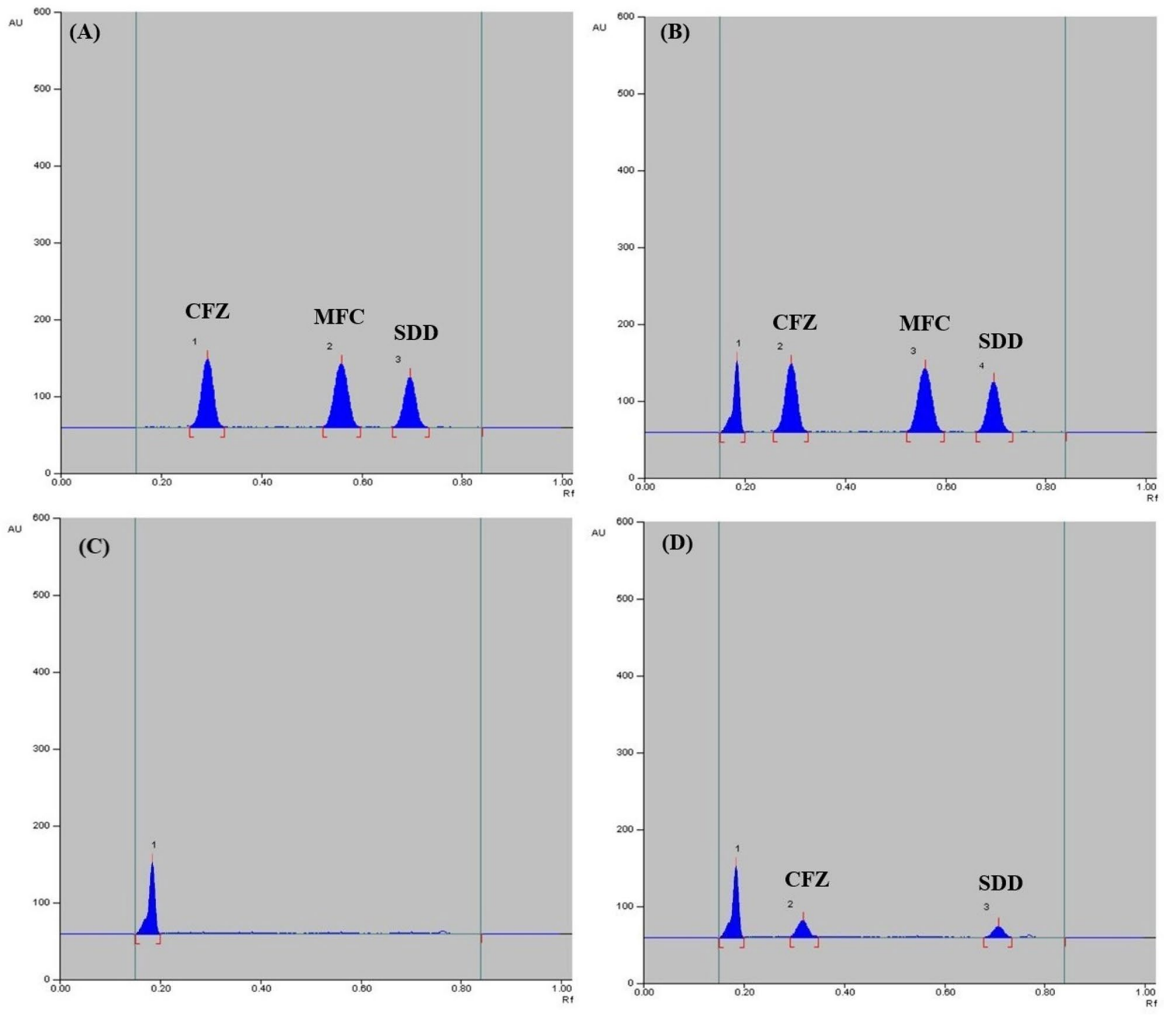
2D-thin layer chromatography-densitogram of (**A**) standard solution (0.04 µg/band) of the studied drugs, (**B**) milk sample spiked with the studied drugs (0.04 µg/band each), (**C**) drug-free milk sample, and (**D**) real milk sample (no.1) showing CFZ and SDD residues.

To assess the specificity of the proposed method, comparative chromatographic analyses were carried out using blank (drug-free) milk samples alongside spiked samples and real milk specimens. As illustrated in Fig. 5, no spectral interferences were observed between endogenous milk components and the target analytes. To minimize matrix-related effects, an efficient sample preparation procedure was applied involving protein precipitation followed by lipid removal using dSPE with EMR-L. This approach significantly reduced potential interferences from complex milk constituents. In addition, detection was carried out at 270 nm, a wavelength at which there is no expected interference from carbohydrates and minerals, further enhancing method selectivity and reducing matrix effects. The components of studied milk samples are shown in Table 6.

**Table 6:** Components of milk samples.

Animal	Water (%)	Protein (%)	Fat (%)	Minerals (%)	Lactose (%)
Cow	85–87	3.2–3.8	3.7–4.4	0.7–0.8	4.8–4.9
Buffalo	82–84	3.3–3.6	7.0–11.5	0.8–0.9	4.5–5.0
Sheep	79–82	5.6–6.7	6.9–8.6	0.8–0.9	4.3–4.8
Goat	87–88	2.9–3.7	4.0–4.5	0.8–0.9	3.6–4.2

#### Robustness

The robustness of the proposed HPTLC-densitometric method was evaluated by systematically investigating the effects of minor, deliberate variations in key operational parameters on the chromatographic response. These modifications were made to assess the method’s ability to withstand typical laboratory fluctuations. The study was conducted by altering the mobile phase composition [ethyl acetate –methanol – triethylamine (50:30:1, v/v/v) ± 0.1 ratio for each component], the detection wavelength (270 ± 1 nm), and the scanning speed (20 ± 2 mm/s). The analytical performance was evaluated based on critical chromatographic metrics: the retention/retardation factor (R_f_), tailing factor (T), and capacity factor (k’).

As summarized in Table 7, the data demonstrated consistent performance across all tested variations, with %RSD values ranging from 0.5 to 1.94 (less than 2%) for all parameters. The R_f_ values remained stable with a variation of less than ± 0.02, T values were consistently below 1.2, and the capacity factor showed minimal deviation. Statistical interpretation of these results indicates that the deliberate variations did not produce statistically significant differences in chromatographic responses (*p* > 0.05, as evidenced by %RSD < 2% for all parameters).

**Table 7:** Robustness results of the proposed HPTLC-densitometric method.

Parameter	Exp. Change	CFZ			MFC			SDD		
		*R*_f_^A^	T ^B^	K^` C^	*R*_f_^A^	T ^B^	K^` C^	*R*_f_^A^	T ^B^	K^` C^
Mobile phase composition (ethyl acetate: methanol: triethylamine, v/v/v)	50.1: 29.9: 0.9 (v/v/v)	0.28	1.06	1.04	0.58	0.98	3.79	0.73	0.99	5.24
	49.9: 30.1: 1.1 (v/v/v)	0.29	1.04	1.01	0.57	0.97	3.82	0.71	1.01	5.26
Detection wavelength (nm)	269	0.29	1.01	1.03	0.59	0.95	3.77	0.74	1.03	5.21
	271	0.28	1.02	1.00	0.57	0.94	3.79	0.72	0.98	5.25
Scanning speed (mm/s)	18	0.28	1.05	1.05	0.59	0.99	3.68	0.73	1.01	5.29
	22	0.29	1.02	1.02	0.58	0.96	3.78	0.75	0.99	5.26
	Mean	0.29	1.03	1.03	0.58	0.97	3.77	0.73	1.00	5.25
	SD	0.01	0.02	0.02	0.01	0.02	0.05	0.01	0.02	0.03
	% RSD	1.92	1.90	1.83	1.54	1.94	1.27	1.94	1.83	0.50

^A^Retardation factor.

^B^Tailing factor.

^C^Capacity factor.

### Application on real milk samples

The developed method was successfully applied to quantify the studied antibiotics in five independent lactating bovines (three buffaloes and two cows) diagnosed with metritis, all of which were receiving the investigated antibiotics at recommended veterinary doses. Following a 72-hour withdrawal period after the final intramuscular administration, a single milk sample (25 mL) was collected from each animal. The samples were analyzed using the described chromatographic conditions. The results are summarized in Table 8. Figure 5 presents the 2D-thin layer chromatographic densitogram of sample 1. All examined samples contained detectable levels of the studied drug residues within the permissible MRLs, except for sample 4, which showed SDD concentration exceeding the corresponding MRL.

The elevated SDD concentration (0.0204 µg/band, exceeding the MRL of 0.01 µg/band) may indicate inadequate withdrawal period compliance, improper dosing, or individual animal metabolic variations. This finding highlights the importance of routine residue monitoring to ensure food safety and regulatory compliance.

This study represents a pilot investigation using five animals to demonstrate method applicability. While the sample size is limited, the primary objective was method development and validation rather than epidemiological surveillance. The results provide proof-of-concept for the analytical approach; larger-scale studies are recommended for population-level residue monitoring.

**Table 8:** Analysis of milk samples using the HPTLC-densitometric method.

Milk samples	Sample type	Found Conc. (µg/band)			MRL in milk (µg/band)		
		CFZ	MFC	SDD	CFZ	MFC	SDD
Sample 1	Buffalo milk	0.0014	–	0.0015	0.005	0.015	0.01
Sample 2	Buffalo milk	–	0.0056	–			
Sample 3	Buffalo milk	0.0025	–	0.0021			
Sample 4	Cow milk	–	–	0.0204			
Sample 5	Cow milk	0.0035	–	–			

### Evaluation of analyte stability in milk samples

The stability of the studied drugs in milk samples stored at −20 °C was evaluated by analyzing spiked milk samples at a concentration of 0.04 µg/band over different storage time points (Normal conditions, 1, 2, and 3 weeks). The mid-concentration level (0.04 µg/band) was selected as representative of typical residue levels near regulatory MRLs. The samples were considered stable if the nominal (actual) concentration remained within ± 15% (calculated as relative error, RE %). RE% is calculated as follows;

$${\text{RE\% = ((Found conc}}{\text{. }} - {\text{nominal conc}}{\text{.)/nominal conc}}{\mathrm{.)*100}}{\mathrm{.}}$$

Results from Table 9 indicated that the RE% values were within ± 15% for up to two weeks of storage, demonstrating the stability of the drugs in milk at −20 °C during this period. However, after three weeks, the RE% values exceeded the specified range, and milk endogenous peaks were observed.

The − 20 °C was selected as it represents standard freezer conditions available in most routine analytical laboratories. While lower temperatures (− 60 °C–− 80 °C) may extend stability, they require specialized equipment and are less practical for routine residue monitoring. The two-week stability at − 20 °C is sufficient for typical sample storage and transport in regulatory workflows.

The observed instability after three weeks is most likely attributed to microbial growth in the milk matrix despite freezing, as evidenced by the appearance of endogenous peaks. Chemical degradation of the analytes is less likely at − 20 °C, but enzymatic activity (β-lactamases from contaminating bacteria) cannot be excluded. The endogenous peaks appeared at Rf values of approximately 0.05–0.15, likely corresponding to free fatty acids and proteolytic degradation products from lipid and protein breakdown. These peaks were not specifically identified as identification was beyond the scope of this stability assessment; however, their interference with analyte quantitation (CFZ Rf = 0.29) was minimal. For routine monitoring, storage at − 20 °C for no more than two weeks is recommended.

**Table 9:** Evaluation of the studied drugs stability in milk samples at storage condition of −20 °C.

Storage time points	CFZ		MFC		SDD	
	(0.04 µg/band)		(0.04 µg/band)		(0.04 µg/band)	
	Found Conc. (µg/band)	RE% ^A^	Found Conc. (µg/band)	RE% ^A^	Found Conc. (µg/band)	RE% ^A^
Normal conditions	0.0396	−1.01	0.0397	−0.83	0.0393	−1.81
After 1 week	0.0385	−3.64	0.0390	−2.53	0.0386	−3.51
After 2 weeks	0.0381	−4.74	0.0382	−4.46	0.0382	−4.44
After 3 weeks	0.0264	−34.10	0.0294	−26.44	0.0303	−24.14

^A^RE% is the relative error percentage.

### Statistical analysis

A comparative statistical assessment between the proposed analytical method and previously reported method^57^ for the target analytes is presented in Table 10. The calculated t- and F-values were lower than their corresponding theoretical limits, indicating that there is no statistically significant difference between the developed method and the reference methods.

**Table 10:** Comparative Statistical Evaluation of the Proposed and Reported Methods for the investigated drugs.

	CFZ		SDD		MFC	
	HPTLC	Published method^57^	HPTLC	Published method^57^	HPTLC	Published method^57^
Mean	99.19	100.39	99.08	96.21	100.28	98.66
± SD	1.23	1.00	0.39	3.53	0.93	0.75
% RSD	0.509	0.482	0.475	0.565	0.331	0.312
Variance	1.512	1	0.152	12.46	0.864	0.562
n	6	6	6	6	6	6
Student’s t- test	1.023 (2.216) ^A^		1.856 (2.216) ^A^		1.963 (2.216) ^A^	
F-test	0.063 (4.69) ^B^		0.052 (4.69) ^B^		0.021 (4.69) ^B^	

^A^Theoretical t-value (0.05) at *n* = 6.

^B^Theoretical F-value (0.05) at n =.

###  The sustainability assessment of the developed method

The sustainability of the developed analytical procedure was systematically evaluated using four assessment tools: the Analytical Eco-Scale^66,67^, Analytical green star area (AGSA)^68^, Blue Applicability Grade Index (BAGI)^69^, and the RGB12 algorithm^70^.

While conventional TLC may offer comparable greenness, HPTLC provides superior resolution, sensitivity, and quantification accuracy (automated application and densitometric scanning), making it the preferred choice for trace residue analysis. The developed method’s greenness score (77 on Eco-Scale) compares favorably with reported TLC methods (typically 70–75), while offering enhanced analytical performance.

The Analytical Eco-Scale approach employs a deductive scoring system (baseline = 100) that penalizes non-green aspects of the methodology, including hazardous reagent usage, energy requirements, waste generation, and operator safety concerns. Based on this evaluation, the developed method achieved an overall Eco-Scale score of 77, as illustrated in Table 11, highlighting the acceptable greenness of our approach.

**Fig. 6:**
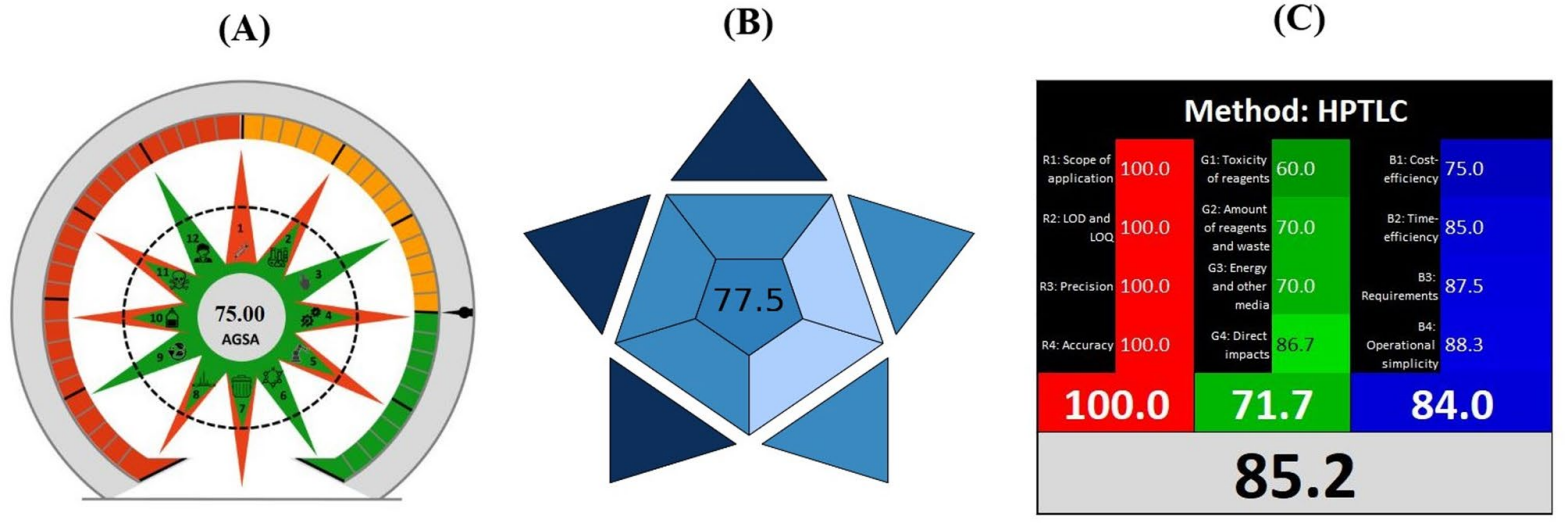
Sustainability assessment of the developed HPTLC-densitometric method using (**A**) AGSA, (**B**) BAGI, and (**C**) RGB12 algorithm.

A complementary Greenness assessment was performed using the Analytical green star area (AGSA) metric, which evaluates compliance with all 12 principles of Green Analytical Chemistry (GAC). AGSA metric is a user-friendly software presents specific multiple-choice questions for each principle, enabling a step-by-step differentiation between methods^68^. Higher sustainability is indicated by higher scores, achieved through minimal sample handling, reduced energy consumption, use of non-toxic reagents, and effective waste management. These scores are cumulative, totaling 36 points (3 points per principle), and are expressed as percentages. The software was utilized to assess the environmental impact of the proposed method, resulting in a greenness score of 75%, indicating a highly environmentally friendly method, as presented in Fig. 6.

The practicality of the developed method was evaluated using the Blue Applicability Grade Index (BAGI)^69^. It offers a standardized, quantitative, and visual tool to assess the analytical method practicality and applicability. This allows laboratories to make well-informed decisions that consider both environmental sustainability and operational efficiency. BAGI evaluates analytical methods based on ten key attributes, such as the type of analysis, the number of analytes simultaneously determined, throughput (number of samples analyzable per hour), reagents and materials used, required instrumentation, the number of samples that can be simultaneously treated, the need for preconcentration, level of automation, the type of sample preparation, and sample quantity needed. The BAGI evaluation is based on four distinct scores that carry equal weight. Each score is associated with a different color shade and contributes to the final score.

The scoring system includes points of 2.5, 5.0, 7.5, and 10, corresponding to white, light blue, blue, and dark blue, respectively. The overall evaluation is represented by an asteroid pictogram with a numerical value at its center. This number reflects the total score of the analytical method and should fall within the range of 25 to 100. A minimum score of 60 points is required for the method to be considered “practical.”

Figure 6 shows the asteroid pictogram evaluation of the developed method, with an overall BAGI score of **77.5**. This score indicates the practicality of the method and its potential for use in a real bioanalytical setting.

The RGB12 model is also utilized to comprehensively assess the method’s green performance (greenness), analytical reliability (redness), and practical applicability (blueness) based on the principles of White Analytical Chemistry (WAC)^70^. Greenness score reflects the environmental impact of the analytical method, including the type and toxicity of reagents used, the amount of chemicals consumed, waste generation, and energy requirements. Redness score reflects the analytical performance and validation quality of the method, and overall applicability of the method to real samples. Blueness score reflects the practical and operational efficiency of the method, including cost-effectiveness, simplicity of the procedure, total analysis time, and overall productivity.

The final RGB12 score is calculated by integrating all the three scores, resulting in a single “whiteness” value that reflects overall method sustainability under the WAC concept. In this study, the developed method achieved a whiteness score of 85.2, as shown in Fig. 6.

**Table 11:** **Comparison of Analytical Eco-Scale for the developed HPTLC method and the reported HPLC method**^57^.

Parameters Reagents	Penalty Points (PPs)	
	The developed HPTLC method	Reported HPLC Method^57^
Type (No. of pictogram and signal word)	Ethyl acetate 2*2 Methanol 3*2 Triethylamine 3*2	Potassium dihydrogen phosphate 0 Methanol 3*2 Acetonitrile 2*2
Amount (hazard* amount)	< 10 ml (16*1 = 16) Total of 16	10–100 ml (12 + 8) Total of 20
Instrument		
Energy consumption	< 1.5 kW/h (1)	< 1.5 kW/h (1)
Emission of vapors or gasses	0	0
Waste		
Waste generated	< 10 ml (3)	> 10 mL (5)
Waste treatment	No treatment (3)	No treatment (3)
Total PPs	23	29
Score (100-PPs)	77	71

## Conclusion

The developed HPTLC–densitometric method provides a reliable and environmentally friendly alternative for the quantitative determination of marbofloxacin, cefazolin, and sulfadimidine in dairy matrices, with effective removal of matrix-related interferences. Compared with conventional HPLC techniques, it offers significant advantages including reduced solvent consumption, lower energy requirements, and simpler operational steps. The chromatographic conditions were systematically optimized and fully validated in accordance with ICH guidelines, ensuring robustness, accuracy, and precision. The method was successfully applied to the analysis of real milk samples collected after the withdrawal period, demonstrating its practical applicability. Comprehensive evaluation further confirmed a favorable environmental profile alongside satisfactory analytical performance, as reflected by improved green chemistry metrics. The proposed method represents a sustainable, efficient, and cost-effective analytical tool suitable for routine regulatory screening laboratories, particularly in resource-limited settings where LC-MS/MS is unavailable. Its simplicity, low cost per sample, and high throughput make it ideal for monitoring veterinary drug residues in milk to ensure compliance with MRLs and protect public health. Overall, the proposed method represents a sustainable, efficient, and cost-effective analytical tool suitable for routine monitoring of veterinary drug residues in milk.

## Data Availability

All data generated or analyzed during this study are included in this published article [and its supplementary information files].
